# A New Method of Inserting an Artificial Crown upon a Root

**Published:** 1879-06

**Authors:** H. A. Moyer

**Affiliations:** Kendallville, Ind.


### A NEW METHOD OF INSERTING AN ARTIFICIAL CROWN UPON A ROOT.

BY H. A. MOYER, KENDALLVILLE, IND.

On looking over my plate register I find that I have made more partial sets of incisors than of any other one kind. The ratio being about three to one, now there is no doubt in my mind that three-fourths of all the cases operated on might have been pivoted, if I could have done so in a permanent manner, and at a moderate charge.

The old way of pivoting with wood pivot answers very well for a time, but soon becomes offensive, and the inevitable result is extraction, and replacement by plate, to the disgust of our patient, who thinks our only object is to make a double fee.

There are ways of pivoting that are considered permanent, but are too complicated, costing more than most patients are willing to pay, and requiring more than ordinary skill to execute in a workmanlike manner. I have endeavored by this method to counteract, to a certain extent, the condemnable practice of extracting roots, that can be made to do good service for years, in the cause of mastication, and thereby obviate the necessity of inserting a plate for one or more teeth.

What I claim for this method is simplicity and ease of manipulations attended with as favorable results as are possible in the more complicated ways.

My manner of proceeding is as follows:

Prepare root as usual for ordinary pivot tooth, by filing or grinding a little below the margin of the gum, then enlarge the nerve canal, making an undercut and a cavity slightly larger on the inside this gives thorough anchorage. A point to be observed in all root fillings, as there is a slight shrinkage in most amalgams, and without a properly shaped cavity, there is danger of the entire filling coming out.

Close the nerve canal with Hill's stopping or any of the preparations of gutta percha bringing it down to bottom of cavity. (The advantage of filling the nerve canal with the gutta percha stopping, is that it can be easily removed, to treat the root should it ever be required). Now fill the root with amalgam, using as little mercury as possible to make the paste, build the filling above the margin of the gum, and dismiss patient allowing ample time for filling to harden. Then trim down with the file allowing filling to overlap the outside edge of root; take an impression of the parts and bite as for plate, hardening plaster cast slightly, (with some dentists this will be considered superfluous, and in a great many cases it is so, but to insure a thoroughly good fit of crown to root, it is necessary. I use red paint in fitting a crown to root, as in fitting a plate to a cast in porcelain work), select a suitable tooth, fit it accurately to the root, then place the artificial crown upon the natural root in its proper place, and with a small probe mark the place for insertion of screw; remove the crown and drill a hole in amalgam filling, corresponding in length and diameter of screw to be used, using a pivot gauge to ascertain the proper depth in filling. Now with mercury soften the amalgam filling slightly on the surface, place crown back in position and with the drive screw it down to its proper place, then fill cavity over head of screw and finish in usual manner.

The teeth used for this kind of work, are to be manufactured for the purpose; the hole for the screw runs through the body of tooth, and has a lining of platinum part way with a thread cut to receive the screw. This platinum lining should be placed in tooth before baking, and is roughened on outside to cause adhesion of the porcelain body to the lining. All who have worked continuous gum will see that it is impossible for the lining to work loose without breaking the tooth.

On the lingual surface there is a properly shaped cavity to be filled over the head of screw and covering all the work. So the only point exposed is the small filling.

The screw used for this purpose is to be made of ten-carat gold wire, with a well defined thread. Although in my judgment any kind of metal sufficiently strong would answer, as it is entirely buried in the surrounding metal.

The only extra instruments necessary for work of this kind are a drill, a drill tap and a screw driver.

The size of the drill, etc., corresponds to number five White's bur gauge, giving sufficient strength to the screw to stand all ordinary mastication. The screws are made in assorted lengths suitable to all kinds of roots.

I have made considerably many experiments in setting pivot teeth. But have made none which have given such perfect satisfaction to my patients as the one above described. Although I have not as yet set one with the platinum lining, my experiments have all been made with the crowns of natural teeth, and with imperfect screws made from brass copper, and iron wire.

I have pivoted some out of the mouth with iron wire, that I could not pull apart with the forceps without breaking the root entirely to pieces. I have endeavored to make this description as plain as possible and trust that this, my favorite method, will meet with a trial.

